# Gene regulatory network and signalling pathway rewiring: How blood cancer cells shift their shapes to evade drug treatment

**DOI:** 10.18632/oncotarget.28662

**Published:** 2024-10-11

**Authors:** Constanze Bonifer, Peter N. Cockerill

**Keywords:** acute myeloid leukaemia, gene regulatory networks, aberrant growth factor signaling, transcription, RUNX1/AP-1 axis

Acute Myeloid Leukemia (AML) is a heterogeneous disease where multiple mutations in genes encoding transcriptional and growth regulators lead to an extensive rewiring of the gene regulatory network (GRN), thereby changing the identity of hematopoietic stem and progenitor cells in a way that blocks myeloid differentiation [1]. One hallmark of AML is the acquisition of mutations in growth factor receptor and growth signalling genes such as FMS-like tyrosine kinase 3 (*FLT3*), the receptor for stem cell factor (*KIT*) and *RAS*. FLT3 is one of the most frequently mutated genes in AML. 25% of AMLs display an internal tandem duplication (ITD) which renders this receptor constitutively active. The prognosis for FLT3-ITD AML patients is dire as even after complete remission after chemotherapy, many patients relapse. Hence, inhibitors that also target the aberrant FLT3 receptor have been introduced but even then most patients relapse and become resistant, by either acquiring additional FLT3 mutations or mutations in downstream signalling molecules. However, many patients do not show additional mutations and rewire their signalling pathways to evade FLT3 inhibition by becoming dependent on other cytokines [2]. Significantly, AML cells can become also become resistant in a non-genetic way by changing their gene expression programs [3] but the role of aberrant signalling in this process remained unclear.

**Figure 1:**
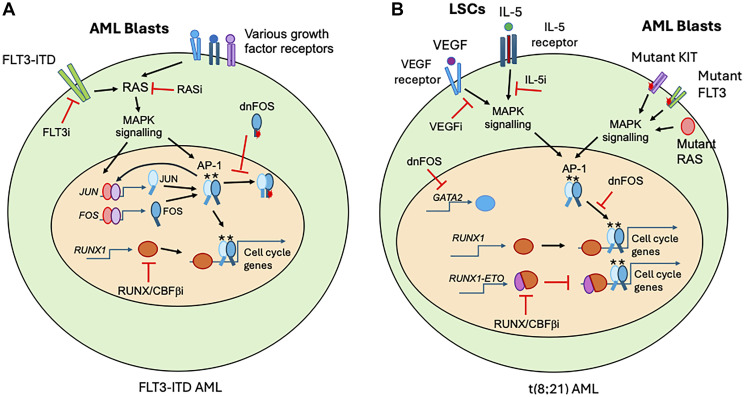
The RUNX1/AP-1 axis operates differently in different types of AML. Cytokine receptors are indicated in different colours with their ligand shown as coloured round shapes on the cell surface. Transcription factors such as JUN and FOS are indicated as elliptical shapes with the DNA binding domains depicted lines below the shape. Gene names are in italics. Asterisks depict phosphorylated proteins. FLT3i, RASi, VEGFi and IL-5i are inhibitors of the respective signalling pathways. RUNX/CBFβi and dnFOS inhibit RUNX1 and AP-1 DNA binding, respectively. The mutant DNA binding domain in dnFOS blocks JUN family members from binding and is shown as a red elliptical shape. Arrows indicate activation, inhibitory action is indicated by a red bar. (**A**) Interplay between signalling and the RUNX1/AP-1 axis in FLT3-ITD AML. (**B**) Interplay between signalling and the RUNX1/AP-1 axis in t(8;21) AML. The RUNX1-ETO fusion protein consists of the RUNX1 DNA binding domain fused to the ETO (RUNX1T1) protein is indication by two shapes fused together.

Recent publications from our group addressed this issue by performing a multi-omics study that investigated how the GRNs in FLT3-ITD patients were rewired as compared to normal cells and in response to FLT3 inhibitor treatment [4, 5]. Several results were noteworthy: (i) mapping of open chromatin regions showed that patients who were initially responsive to FLT3 inhibition strongly rewired their GRNs and formed many new connection patterns between transcription factors (TFs) and their target genes, whereas the non-responsive patient did not; (ii) chromatin immunoprecipitation (ChIP) experiments showed that drug treatment led to a loss of binding of RUNX1, the master regulator of hematopoiesis, and the MAP-Kinase (MAPK) inducible TF AP-1; (iii) the abolition of AP-1 binding by a dominant-negative version of the TF (dnFOS) abolished RUNX1 binding at hundreds of binding sites as well, demonstrating that RUNX1 binding is AP-1 dependent, and finally (iv) that both AP-1 and RUNX1 inhibition led to a profound cell cycle block as summarised in Figure 1A.

How do the cells overcome the cell cycle block during relapse? It turned out that FLT3 inhibitor treatment upregulated multiple signalling genes (such as *KIT*), many of which were RUNX1 and AP-1 targets. We therefore speculated that such upregulation primed the cells for bypassing FLT3 signal inhibition using other cytokines. Indeed, when cytokines such as IL-3, GM-CSF or FGF2 were added to patient cells growing in culture, AML cell growth was restored even in the presence of FLT3 inhibitors. With IL-3, the binding of RUNX1 to chromatin was also restored, demonstrating that MAPK pathways can indeed be rewired and re-activated by alternative cyotines. However, most MAPK signalling is transmitted via RAS (Figure 1A), which also frequently acquires activating mutations in either presentation or relapse patients. To test the dependency upon RAS in activating alternative signalling pathways, we targeted FLT3 inhibitor resistant cells with a pan-RAS inhibitor which inhibits both mutated and unmutated RAS [6]. This treatment completely abrogated FLT3 and IL-3 dependent RUNX1 binding and cellular growth both *in vitro* and *in vivo* [4]. Our data show that the RUNX1/AP-1 axis is a highly flexible genomic target which links proliferation to differentiation and integrates tissue-specific TF activity with growth signalling. Inhibition of either factor leads to cell cycle arrest and a block in differentiation. AP-1 is a common node in all AML-specific GRNs we have analysed so far and blocking its activity completely abrogates tumour formation in two different (FLT3-ITD and t(8;21) xenograft AML models [1]. One of the most fascinating results of our recent work is the finding that the RUNX1/AP-1 axis can be activated in different ways, depending on the AML sub-type. In t(8;21) AML, which expresses the oncogene RUNX1-ETO, an elaborate balance between RUNX1 and this oncogene together with AP-1 regulates cell cycle progression (Figure 1B). Moreover, different signalling pathways are used to activate growth in quiescent GATA2 expressing leukemic stem cells (LSCs) and proliferating AML blast cells. To kick-start LSC growth and down-regulate *GATA2*, cells hijack lineage inappropriate signalling pathways, including endothelial VEGF and eosinophilic IL-5 signalling that again target the interdependent RUNX1/AP-1 axis [7]. Conversely, AML blast cells display a different growth phenotype, which is driven by mutant and constitutively active versions of KIT, RAS and FLT3 (Figure 1B).

In summary, drugs that target individual signalling pathways in AML often fail to stop proliferation malignant growth, due to the wide variety, redundancy and cross talk between multiple pathways regulating and differentiation. Moreover, signalling pathways can be rewired via the use of alternative cytokines and receptors. We therefore need to consider identifying AML-subtype-specific GRNs that drive cancer phenotypes and target aberrantly active TFs such as AP-1 and RUNX1 directly. Combined with refined delivery methods targeting specific cells we believe that with this strategy AML can be eliminated.
